# On the System of Diophantine Equations *x* ^2^ − 6*y* ^2^ = −5 and *x* = *az* ^2^ − *b*

**DOI:** 10.1155/2014/632617

**Published:** 2014-06-17

**Authors:** Silan Zhang, Jianhua Chen, Hao Hu

**Affiliations:** ^1^School of Mathematics and Statistics, Wuhan University, Hubei 430072, China; ^2^College of Science, Huazhong Agricultural University, Hubei 430070, China

## Abstract

Mignotte and Pethö used the Siegel-Baker method to find all the integral solutions (*x*, *y*, *z*) of the system of Diophantine equations *x* ^2^ − 6*y* ^2^ = −5 and *x* = 2*z* ^2^ − 1. In this paper, we extend this result and put forward a generalized method which can completely solve the family of systems of Diophantine equations *x* ^2^ − 6*y* ^2^ = −5 and *x* = *az* ^2^ − *b* for each pair of integral parameters *a*, *b*. The proof utilizes algebraic number theory and *p*-adic analysis which successfully avoid discussing the class number and factoring the ideals.

## 1. Introduction

Let *Z*, *N*, and *Q* be the sets of all integers, positive integers, and rational numbers, and let *a*, *b* be the integers. The system of Diophantine equations

(1) $$\begin{array}{c} x^{2}-6y^{2}=-5,\;\;x=az^{2}-b \end{array}$$

is a quartic model of an elliptic curve that has been investigated in many papers. Mignotte and Pethő [1] used the Siegel-Baker method to solve (1) for *a* = 2 and *b* = 1; however, their method was complicated as a combination of algebraic and transcendental number theory. In 1998, Cohn [2] gave an elementary proof of the above system of equations for *a* = 2 and *b* = 1. In 2004, Le [3] used a similar elementary method to extend the result of Cohn's work and proposed an effective method solving the system of equations

(2) $$\begin{array}{c} x^{2}-Dy^{2}=1-D,\;\;x=2z^{2}-1, \end{array}$$

where *D* − 1 is the power of an odd prime. As an example, solutions of the equations for *D* = 6 and *D* = 8 are given in the paper so as to show the effectiveness of the method.

In this paper, we use algebraic number theory and Skolem's *p*-adic method [4] to solve (1), and the method is relatively simple. In the proposed method, both the consideration of the class number in the field and the factorization of ideals of integral ring are avoided. Moreover, a faster algorithm proposed in [5] to compute the fundamental unit and the set of nonassociated factors is used.

In order to well interpret the main result, the symbol notation used in this paper is defined as below.

Here, we assume that *m* ≥ 0 is an integer and $\varepsilon_{0}=5+2\sqrt{6}$ is the fundamental unit in the field $Q(\sqrt{6})$, and let *A* denote $\left(\beta \varepsilon_{0}^{m}+\bar{\beta }{\bar{\varepsilon_{0}}}^{m}\right)/\left(\beta +\bar{\beta }\right)$, where *β* ^2^ = ±*ηε*_0_ or *β* ^2^ = ±*η*, with $\eta =1+\sqrt{6}$; ${\bar{\beta }}^{2}$ denotes the conjugate of *β* ^2^ in $Q(\sqrt{6})$.

The main result of this paper is as follows.

**Theorem 1 .**

Let (*x*, *y*, *z*) be an integral solution of (1). Then *z* exists only when it satisfies one of the following four equations for *k* ∈ *Z*:

(*N*1) 2*az* + *θA* = ±*αε* ^*k*^ with $\theta^{2}=2a(1-\sqrt{-5})$,
(*N*2) 2*az* + *θA* = ±*αε* ^*k*^ with $\theta^{2}=4a(1+\sqrt{-5})$,
(*N*3) 2*az* + *θA* = ±*αε* ^*k*^ with $\theta^{2}=2a(-1-\sqrt{-5})$,
(*N*4) 2*az* + *θA* = ±*αε* ^*k*^ with $\theta^{2}=4a(-1+\sqrt{-5})$,

where *ε* is the fundamental unit of the totally complex quartic field of *Q*(*θ*), *α* is the nonassociated factor such that $\alpha \alpha_{-}=4a(b-\sqrt{-5})$, *α*_−_ denotes the relative conjugate of *α*, and *A* is referred to above.

As the application to the theorem, we give the following corollary.

**Corollary 2 .**

The system of (1) for *a* = 2 and *b* = 1 has exactly six integral solutions: (*x*, *y*, *z*) = (16561, ±6761, ±91), (71, ±29, ±6), (17, ±7, ±3), (7, ±3, ±2), (1, ±1, ±1), and (−1, ±1,0).

## 2. Proof of the Theorem

Before the proof of the theorem, Lemma 3 is needed.

**Lemma 3 .**

If *m*, *ε*_0_, *β*, and $\bar{\beta }$ are defined as before, then $A=\left(\beta \varepsilon_{0}^{m}+\bar{\beta }{\bar{\varepsilon_{0}}}^{m}\right)/\left(\beta +\bar{\beta }\right)$ is an algebraic number in the field $Q(\sqrt{-5})$.

**Proof**

Rewriting *A*, we have

(3) A=βε0m+β¯ε0¯mβ+β¯=(βε0m+β¯ε0¯m)(β+β¯)(β+β¯)2=β2ε0m+β¯2ε0¯m+ββ¯(ε0m+ε0¯m)β2+β¯2+2ββ¯.

Since $\beta^{2}+{\bar{\beta }}^{2}$, $\beta^{2}\varepsilon_{0}^{m}+{\bar{\beta }}^{2}{\bar{\varepsilon_{0}}}^{m}$, and $\varepsilon_{0}^{m}+{\bar{\varepsilon_{0}}}^{m}$ are all rational integers and $\beta \bar{\beta }=\sqrt{-5}$, then clearly *A* is an algebraic number. Thus, the lemma is proven.

**Proof of Theorem.**

There are four separate cases in consideration during the proof. Since the process is very similar in each case, some details will be omitted for simplicity. Now we prove the theorem.

After rewriting the first equation of (1), factorization in the field $Q(\sqrt{6})$ yields

(4) $$\begin{array}{c} \left(x+\sqrt{6}y\right)\left(x-\sqrt{6}y\right)=\left(1+\sqrt{6}\right)\left(1-\sqrt{6}\right). \end{array}$$

Then we have

(5) (x+6y)=±ηε0n,(x−6y)=±η¯ε0¯n, with  n∈Z.

Adding (5), we get

(6) $$\begin{array}{c} 2x=\pm \left(\eta \varepsilon_{0}^{n}+\bar{\eta }{\bar{\varepsilon_{0}}}^{n}\right). \end{array}$$

The solution of (6) is split into four cases.

*Case 1.* Assume that *n* = 2*m* + 1 is odd and $2x=\eta \varepsilon_{0}^{n}+\bar{\eta }{\bar{\varepsilon_{0}}}^{n}$. Then

(7) 2x=ηε02m+1+η¯ε0¯2m+1=(β+β¯)2(βε0m+β¯ε0¯mβ+β¯)2−2ββ¯,with  β2=ηε0.

Since

(8) $$\begin{array}{c} {\left(\beta +\bar{\beta }\right)}^{2}=34+2\sqrt{-5}=\left(1-\sqrt{-5}\right){\left(3+\sqrt{-5}\right)}^{2},\\ \beta \bar{\beta }=\sqrt{-5}, \end{array}$$

by inserting (7) and the second equation of (1) into (6), we get

(9) $$\begin{array}{c} 2az^{2}-\theta^{2}A^{2}=2\left(b-\sqrt{-5}\right), \end{array}$$

where $\theta^{2}=1-\sqrt{-5}$ and $A=(3+\sqrt{-5})(\left(\beta \varepsilon_{0}^{m}+\bar{\beta }{\bar{\varepsilon_{0}}}^{m}\right)/\left(\beta +\bar{\beta }\right))$. We multiply both sides of the equation by 2*a* to obtain

(10) $$\begin{array}{c} {\left(2az\right)}^{2}-\theta^{2}A^{2}=4a\left(b-\sqrt{-5}\right)=\alpha \alpha_{-}. \end{array}$$

Without loss of generality, we also denote $\theta^{2}=2a(1-\sqrt{-5})$ and $A=(3+\sqrt{-5})(\left(\beta \varepsilon_{0}^{m}+\bar{\beta }{\bar{\varepsilon_{0}}}^{m}\right)/\left(\beta +\bar{\beta }\right))$. Factoring (9) in the field *Q*(*θ*), we have

(11) $$\begin{array}{c} \left(N1\right)\;2az+\theta A=\pm \alpha \varepsilon^{k},\;\text{with}\;\;\theta^{2}=2a\left(1-\sqrt{-5}\right),\;\;k\in Z. \end{array}$$

*Case 2.* In another case, when  *n* = 2*m* is even and $2x=\eta \varepsilon_{0}^{n}+\bar{\eta }{\bar{\varepsilon_{0}}}^{n}$, then

(12) 2x=ηε02m+η¯ε0¯2m=(β+β¯)2(βε0m+β¯ε0¯mβ+β¯)2−2ββ¯,with  β2=η.

Similarly, we have

(13) $$\begin{array}{c} {\left(2az\right)}^{2}-\theta^{2}A^{2}=4a\left(b-\sqrt{-5}\right)=\alpha \alpha_{-}, \end{array}$$

where $\theta^{2}=4a(1+\sqrt{-5})$ and $A=\left(\beta \varepsilon_{0}^{m}+\bar{\beta }{\bar{\varepsilon_{0}}}^{m}\right)/\left(\beta +\bar{\beta }\right)$. Furthermore, we know that *z* satisfies

(14) $$\begin{array}{c} \left(N2\right)\;2az+\theta A=\pm \alpha \varepsilon^{k}\;\text{with}\;\;\theta^{2}=4a\left(1+\sqrt{-5}\right),\;\;k\in Z. \end{array}$$

*Case 3. *Consider *n* = 2*m* + 1 is odd and $2x=-(\eta \varepsilon_{0}^{n}+\bar{\eta }{\bar{\varepsilon_{0}}}^{n})$. By the same consideration, we deduce that *z* satisfies

(15) $$\begin{array}{c} \left(N3\right)\;2az+\theta A=\pm \alpha \varepsilon^{k}\;\text{with}\;\;\theta^{2}=2a\left(-1-\sqrt{-5}\right),\;\;k\in Z, \end{array}$$

where $A=(3-\sqrt{-5})(\left(\beta \varepsilon_{0}^{m}+\bar{\beta }{\bar{\varepsilon_{0}}}^{m}\right)/\left(\beta +\bar{\beta }\right))$.

*Case 4. *Consider *n* = 2*m* is even and $2x=-(\eta \varepsilon_{0}^{n}+\bar{\eta }{\bar{\varepsilon_{0}}}^{n})$. Similarly, we know that *z* satisfies

(16) $$\begin{array}{c} \left(N4\right)\;2az+\theta A=\pm \alpha \varepsilon^{k}\;\text{with}\;\;\theta^{2}=4a\left(-1+\sqrt{-5}\right),\;\;k\in Z, \end{array}$$

where $A=\left(\beta \varepsilon_{0}^{m}+\bar{\beta }{\bar{\varepsilon_{0}}}^{m}\right)/\left(\beta +\bar{\beta }\right)$.

Thus, we complete the proof of the theorem.

## 3. Proof of the Corollary

**Remark 4 .**

The method of the proof of the corollary is a special instance of general procedure for the computation of integral points on some quartic model of elliptic curves. The method is relatively simple, because it avoids the use of class number and ideal factorization in imaginary quartic fields.

Before the proof, we give the following lemma.

**Lemma 5 .**

For any integer *m* ≠ 0 and *i* > 1, one has $V_{p}(p^{i}\left(\begin{array}{c} m\\ i \end{array}\right))>V_{p}(p\left(\begin{array}{c} m\\ 1 \end{array}\right))$, where *V*_*p*_(·) denotes the standard *p*-adic valuation and *p* is an odd prime.

**Proof**

We know

(17) Vp(pi(mi))−Vp(p(m1))  =i−1+Vp(mi)−Vp(m1)  =i−1+Vp((m−1)⋯(m−i+1)i!)  =i−1+Vp((m−1)⋯(m−i+1))−Vp(i!)  ≥i−1−Vp(i!)  =i−1−([ip]+[ip2]+⋯)  ≥i−1−ip−1  ≥(p−2p−1)i−1>0, with  i>1.

Therefore, for *i* > 1, the *p*-adic valuation of the term of $p^{i}\left(\begin{array}{c} m\\ i \end{array}\right)$ exceeds the *p*-adic valuation of the term of $p\left(\begin{array}{c} m\\ 1 \end{array}\right)$. Thus, we complete the proof of Lemma 5.

To complete the proof of the corollary, the fundamental unit in the totally complex quartic field *Q*(*θ*) is computed. Furthermore, nonassociated factor of $2-2\sqrt{-5}$ in the ring of integers of *Q*(*θ*) is also calculated. The idea of computation of fundamental unit and nonassociated factorization stems from Zhu and Chen [5, 6] and Buchmann's work [7], which offered a fast implementation scheme. Results are obtained via MATHEMATICA 7.0, which are listed in Table 1.

**Proof**

*Case 1. *Substituting *a* = 2 and *b* = 1 into (9), we know that *z* satisfies

(18) $$\begin{array}{c} 4z^{2}-\theta^{2}A^{2}=2-2\sqrt{-5}=\alpha \alpha_{-}. \end{array}$$

Equation* (N*1) is reduced to

(19) $$\begin{array}{c} \left(N5\right)\;2z+\theta A=\pm \alpha \varepsilon^{k}\;\text{with}\;\;\theta^{2}=1-\sqrt{-5},\;\;k\in Z. \end{array}$$

From Table 1, we get *ε* = 11 + 2*θ* − 6*θ* ^2^ − 4*θ* ^3^ and *α* = 6 + 4*θ* − *θ* ^3^. From Lemma 3, we know that *A* is an element in the field $Q(\sqrt{-5})$. If we expand 2*z* + *θA* in the basis 1, *θ*, *θ* ^2^, and *θ* ^3^ of *Q*(*θ*), we obtain 2*z* + *θA* = *a* + *bθ* + *cθ* ^2^ + *dθ* ^3^  (*a*, *b*, *c*, *d* ∈ *Q*); then, from Lemma 3, we also know that *c* = 0. In short, we denote this fact by (2*z* + *θA*)_2_ = 0.

To use the *p*-adic analysis, a suitable prime *p* is needed. Here, we take *p* = 7. A straightforward computation shows that

(20) $$\begin{array}{c} \varepsilon^{8}\equiv 1\left(\mathrm{mod}\;7\right). \end{array}$$

Since (*αε* ^0^)_2_ ≡ 0(mod⁡ 7), (*αε* ^7^)_2_ ≡ 0(mod⁡ 7), and (*αε* ^*i*^)_2_≢0(mod⁡ 7) (*i* = 2,…, 6), we get *k* ≡ 0,7(mod⁡ 8).

(i) Let *k* ≡ 0(mod⁡ 8); since *ε* ^8^ ≡ 1(mod⁡ 7), we obtain *ε* ^8^ = 1 + 7*ξ*; then

(21) ±(2z+θA)=α(1+7ξ)m=α+7(m1)(αξ) +72(m2)(αξ2)+⋯+7m(mm)(αξm),

with *k* = 8*m*. So we have

(22) $$\begin{array}{c} \pm {\left(2z+\theta A\right)}_{2}={\left(\alpha +7\left(\begin{array}{c} m\\ 1 \end{array}\right)\left(\alpha \xi \right)+\cdots +7^{m}\left(\begin{array}{c} m\\ m \end{array}\right)\left(\alpha \xi^{m}\right)\right)}_{2}. \end{array}$$

From Lemma 5 and (*αξ*)_2_ = (−30 + 30*θ* + 60*θ* ^2^ + 30*θ* ^3^)_2_≢0(mod⁡ 7), we get *m* = 0 and *z* = ±3 by working modulo 7^*r*+2^ on formula (13), where 7^*r*^||*m*.

(ii) When *k* ≡ 7(mod⁡ 8), we have

(23) ±(2z+θA)=αε−1(1+7ξ)m=αε−1+7(m1)(αε−1ξ) +⋯+7m(mm)(αε−1ξm),

with *k* = −1 + 8*m*. So we have

(24) ±(2z+θA)2=(αε−1+7(m1)(αε−1ξ) +⋯+7m(mm)(αε−1ξm))2.

Similar deduction shows that *m* = 0 and *z* = ±68.

*Case 2.* Secondly, we similarly consider the following equation:

(25) $$\begin{array}{c} \left(N6\right)\;2z+\theta A=\pm \alpha \varepsilon^{k},\;\text{with}\;\;\theta^{2}=2+2\sqrt{-5},\;\;k\in Z. \end{array}$$

From Table 1, we get *ε* = −49 − 20*θ* + 3*θ* ^2^ + (7/2)*θ* ^3^ and *α* = 2 + *θ*. By the same argument we choose *p* = 23 and *ε* ^11^ ≡ 1(mod⁡ 23). Similarly we have *k* ≡ 0,1(mod⁡ 11); then we can deduce *z* = ±1 and *z* = ±91, respectively.

*Case 3.* Consider the following equation:

(26) $$\begin{array}{c} \left(N7\right)\;2z+\theta A=\pm \alpha \varepsilon^{k}\;\text{with}\;\;\theta^{2}=-1-\sqrt{-5},\;\;k\in Z. \end{array}$$

We also have *ε* = −85 + 34*θ* + 30*θ* ^2^ + 38*θ* ^3^, *α* = 4 + *θ* ^3^, *p* = 5, and *ε* ^4^ ≡ 1(mod⁡ 5). Direct deductions show that *z* = ±2 and *z* = ±11798.

*Case 4.* The final equation is

(27) $$\begin{array}{c} \left(N8\right)\;2z+\theta A=\pm \alpha \varepsilon^{k}\;\text{with}\;\;\theta^{2}=-2+2\sqrt{-5},\;\;k\in Z. \end{array}$$

It corresponds to *ε* = 1 − 2*θ* − *θ* ^2^ − (1/2)*θ* ^3^, *α* = 12 − *θ*, *p* = 13, and *ε* ^5^ ≡ 1(mod⁡ 13). A similar deduction yields *z* = 0 and *z* = ±6.

All in all, if (*x*, *y*, *z*) is an integral solution of (1),

(28) $$\begin{array}{c} \pm z=3,68,1,91,2,11798,0,6. \end{array}$$

Substituting (24) into the system of Diophantine equations (1), we get all integral solutions, namely, (*x*, *y*, *z*) = (16561, ±6761, ±91), (71, ±29, ±6), (17, ±7, ±3), (7, ±3, ±2), (1, ±1, ±1), and (−1, ±1,0).

This completes the proof of the corollary.

**Remark 6 .**

Like before, we can solve a family of systems of Diophantine equations (1). As a direct application of this theorem, systems of equations with parameters 1 ≤ *a* ≤ 10 and 1 ≤ *b* ≤ 10 are solved and results are listed in Table 2. For simplicity, we only list the positive *z*-value of solutions (*x*, *y*, *z*).

## Figures and Tables

**Table 1:** Associated number field.

θ^2^	Integral basis	Fundamental unit	Nonassociated factor of $2-2\sqrt{-5}$
$1-\sqrt{-5}$	1, θ, θ^2^, θ^3^	11 + 2θ − 6θ^2^ − 4θ^3^	6 + 4θ − θ^3^
$2+2\sqrt{-5}$	$1,\theta ,\frac{1}{2}\theta^{2},\frac{1}{4}\theta^{3}$	$-49-20\theta +3\theta^{2}+\frac{7}{2}\theta^{3}$	2 + θ
$-1-\sqrt{-5}$	1, θ, θ^2^, θ^3^	−85 + 34θ + 30θ^2^ + 38θ^3^	4 + θ^3^
$-2+2\sqrt{-5}$	$1,\theta ,\frac{1}{2}\theta^{2},\frac{1}{4}\theta^{3}$	$1-2\theta -\theta^{2}-\frac{1}{2}\theta^{3}$	12 − θ

**Table 2:** Positive *z*-value of the solution.

	*a* = 1	*a* = 2	*a* = 3	*a* = 4	*a* = 5	*a* = 6	*a* = 7	*a* = 8	*a* = 9	*a* = 10
*b* = 1	*z* = 0	*z* = 0,1, 2, 3,6, 91	*z* = 0	*z* = 0	*z* = 0	*z* = 0	*z* = 0	*z* = 0,1, 3	*z* = 0	*z* = 0
*b* = 2	*z* = 1,3	NS	*z* = 1	NS	NS	NS	NS	NS	*z* = 1	NS
*b* = 3	*z* = 2	*z* = 1,59	NS	*z* = 1	*z* = 2	NS	NS	NS	NS	*z* = 1,83
*b* = 4	NS	NS	*z* = 1,5	NS	*z* = 1	NS	NS	NS	NS	NS
*b* = 5	*z* = 2	NS	*z* = 2	*z* = 1	NS	*z* = 1	NS	NS	NS	NS
*b* = 6	NS	NS	NS	NS	*z* = 1	NS	*z* = 1,5	NS	NS	NS
*b* = 7	*z* = 0	*z* = 0,2	*z* = 0	*z* = 0	*z* = 0	*z* = 0,1, 2	*z* = 0	*z* = 0,1	*z* = 0	*z* = 0
*b* = 8	*z* = 1,3, 5,41	NS	NS	NS	NS	NS	*z* = 1	NS	*z* = 1	NS
*b* = 9	*z* = 4	*z* = 1,2, 29	NS	*z* = 2	*z* = 4	NS	NS	*z* = 1	NS	*z* = 1
*b* = 10	*z* = 3,9	NS	*z* = 1,3	NS	NS	NS	NS	NS	*z* = 1,3	NS

“NS” refers to “no solution.”
